# A Rosenzweig-MacArthur (1963) Criterion for the Chemostat

**DOI:** 10.1155/2016/5626980

**Published:** 2016-07-18

**Authors:** Torsten Lindström, Yuanji Cheng

**Affiliations:** ^1^Department of Mathematics, Linnaeus University, 35195 Växjö, Sweden; ^2^School of Technology, Malmö University, 20506 Malmö, Sweden

## Abstract

The Rosenzweig-MacArthur (1963) criterion is a graphical criterion that has been widely used for elucidating the local stability properties of the Gause (1934) type predator-prey systems. It has not been stated whether a similar criterion holds for models with explicit resource dynamics (Kooi et al. (1998)), like the chemostat model. In this paper we use the implicit function theorem and implicit derivatives for proving that a similar graphical criterion holds under chemostat conditions, too.

## 1. Introduction 

Several ecological phenomena are studied under chemostat conditions; compare Smith and Waltman [1]. A phenomenological model containing such a situation is given by

(1) s˙=CD−Ds−axs1+abs,x˙=amxs1+abs−Dx−Axy1+ABx,y˙=AMxy1+ABx−Dy.

Here *s* > 0 is the substrate, *x* > 0 is the prey having the substrate *s* as its limiting resource, and *y* > 0 is a predator feeding on the prey *x*. The parameters *C* > 0, *D* > 0, *a* > 0, *b* > 0, *m* > 0, *A* > 0, *B* > 0, and *M* > 0 stand for concentration, dilution rate, search rate for the prey, handling time for the prey (cf. [2]), conversion factor for the prey, search rate for the predator, handling time for the predator, and conversion factor for the predator, respectively.

A reduction into two dimensions [3] is often made when studying (1) and related systems. More precisely, consider the function

(2) $$\begin{array}{c} H_{1}\left(\;s,x,y\;\right)=ms+x+\frac{y}{M}-mC. \end{array}$$

It satisfies ${\dot{ℋ}}_{1}=-D{ℋ}_{1}$ meaning that the surface *ℋ*_1_ = 0 is asymptotically invariant for (1). A study of the system on this surface allows for reducing it to a planar predator-prey system as follows:

(3) x˙=axmC−x−y/M1+ab/mmC−x−y/M−Dx−Axy1+ABx,y˙=AMxy1+ABx−Dy.

Such reductions can be made rigorously under certain conditions; see Smith and Waltman [1]. As an example for how such a procedure may break down Thieme [4] gave the following example in cylindrical coordinates *r*, *θ*, *x*_3_, *x*_1_ = *r* cos *θ*, *x*_2_ = *r* sin *θ*:

(4) r˙=r1−r,θ˙=βrsin⁡θ+x3,x˙3=−γx3,

with initial data 0 < *r*(0) < 1, 0 ≤ *θ* < 2*π*, *x*_3_(0) ≤ 0 and positive constant parameters. In this case, consider the function

(5) $$\begin{array}{c} H_{2}=x_{3}^{2}. \end{array}$$

Also here, we have

(6) $$\begin{array}{c} {\dot{H}}_{2}=-2\gamma x_{3}^{2}=-2\gamma H_{2}<0 \end{array}$$

meaning that the surface *ℋ*_2_ = 0 is asymptotically invariant for (4) and a study of (4) on this surface should allow for reducing it to the planar system

(7) r˙=r1−r,θ˙=βrsin⁡θ.

**Figure 1:**
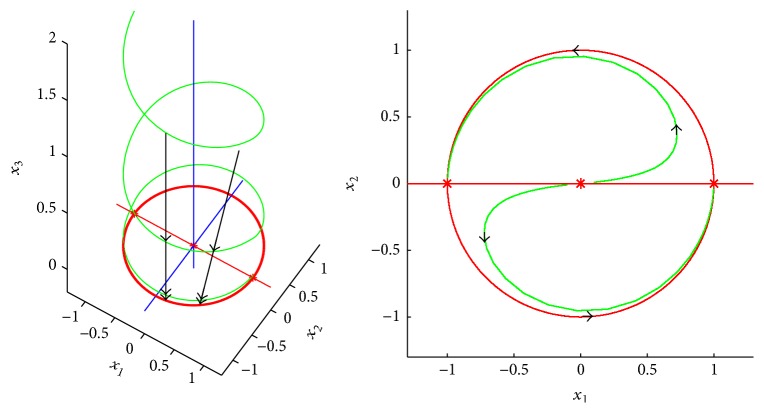
Illustration of the geometry and limit sets in Thieme's [4] example (4).

In Cartesian coordinates, (4) has three equilibria (0,0, 0), (1,0, 0), (−1,0, 0) that are illustrated by *∗*-marks in Figure 1. All solutions are attracted towards the unit circle. Solutions with initial conditions in the plane *x*_3_ = 0 has some equilibrium on the unit circle as its limit set. But if *x*_3_(0) > 0, then the limit set is the whole unit circle. We see, however, that the chain recurrent set [5] is the whole unit circle (and the origin) and does not depend on initial conditions.

Back to (3), if *b* ≠ 0, then the growth function of (3) is given by

(8) hxaxmC−x1+ab/mmC−x−Dx=m/b−Dx+m2ab2−m2/ab21+abCab/mm/ab+mC−x

in the absence of predators. Therefore, growth function is unimodal on the interval

(9) $$\begin{array}{c} \left[\;0,\frac{amC\left(1-D/\left(m/b\right)\right)-D}{a\left(1-D/\left(m/b\right)\right)}\;\right] \end{array}$$

provided

(10) $$\begin{array}{c} D<\frac{amC}{1+abC}<\frac{m}{b}. \end{array}$$

The last inequality is identically true. We shall later use the results above to introduce relevant coordinate-transformations for (3).

Equivalents of the predator-prey system (3) have been studied in Smith and Waltman [1] and Kuang [6]. The results were that local stability implies global stability and that uniqueness of limit cycles was proved for a certain range of parameters. It is still not known whether the limit cycle is unique for all parameters of (3) and a further analysis and improvement of these results remain outside the scope of this paper.

## 2. A Related Gause [7] Type Predator-Prey System

We start this study by relating (3) to a widely used class of predator-prey systems. Assuming that *b* = 0, we get

(11) x˙=axmC−x−Dx−axyM−Axy1+ABx,y˙=AMxy1+ABx−Dy

and this model can be identified as a Gause [7] type predator-prey model on the isocline form

(12) x˙=fxFx−y,y˙=yψx,

with

(13) Fx=axmC−x−Dxax/M+Ax/1+ABx,fx=axM+Ax1+ABx,ψx=AMx1+ABx−D;

see Lindström and Cheng [8]. In general, the conditions on the involved functions are stated as

(A-I) *f*, *ψ*, and *F* are *C* ^1^([0, *∞*)),
(A-II) *f*(0) = 0, *f*(*x*) > 0 for *x* > 0,
(A-III) (*x* − 1)*F*(*x*) < 0 for *x* ≠ 1,
(A-IV) (*x* − *λ*_1_)*ψ*(*x*) > 0, *x* ≠ *λ*_1_ > 0,

and it is easy to see that functions (13) meet criteria (A-I)–(A-IV) and that the solution of *ψ*(*x*) = 0 (the predator isocline) in this case gives

(14) $$\begin{array}{c} \lambda_{1}=\frac{D}{A\left(M-BD\right)}. \end{array}$$

If (A-I)–(A-IV) and 0 < *λ*_1_ < 1, then solutions of system (12) remain positive and bounded [8]. Moreover, it has three equilibria: (0,0) which is a saddle, (1,0) also a saddle, and finally (*λ*_1_, *F*(*λ*_1_)) that has the Jacobian

(15) $$\begin{array}{c} J\left(\;\lambda_{1},F\left(\;\lambda_{1}\;\right)\;\right)=\left(\begin{array}{cc} f\left(\lambda_{1}\right)F^{\prime }\left(\lambda_{1}\right) & -f\left(\lambda_{1}\right)\\ \lambda_{1}\psi^{\prime }\left(\lambda_{1}\right) & 0 \end{array}\right). \end{array}$$

We have det *J*(*λ*_1_, *F*(*λ*_1_)) = *λ*_1_ *f*(*λ*_1_)*ψ*′(*λ*_1_) > 0 and Tr *J*(*λ*_1_, *F*(*λ*_1_)) = *f*(*λ*_1_)*F*′(*λ*_1_) with *f*(*λ*_1_) > 0, so the Trace-determinant criterion [9] gives immediately the classical Rosenzweig-MacArthur [10] criterion stating that the interior equilibrium is locally asymptotically stable when the predator isocline (*x* = *λ*_1_) intersects the prey isocline (*y* = *F*(*x*)) at point where the prey isocline decreases *F*′(*λ*_1_) < 0 and is unstable when *F*′(*λ*_1_) > 0. In fact, all the topological properties including results of global stability and uniqueness of limit cycles for all parameters of (11) are known; see Lindström and Cheng [8].

## 3. Reparametrization

We reparameterize the system in order to eliminate some of the parameters involved (see, e.g., [11]). We remember the growth interval (9) and introduce the new variables,

(16) ξ=a1−Db/mamC1−Db/m−Dx,η=a1−Db/mMamC1−Db/m−Dy,τ=amC1−Db/m−Dt,

and the new parameters,

(17) α=MAa1−Db/m,β=ABa1−Db/mamC1−Db/m−D,κ=bm·amC1−Db/m−D1−Db/m,γ=abC,μ=MA1−DB/Ma1−Db/m,λ2=D/amC1−Db/m−DMA1−DB/M/a1−Db/m.

Now, (3) takes the form

(18) ξ˙=ξ1−ξ−η1+γ−κξ+η−ηαξ1+βξ,η˙=ημξ−λ21+βξ,

with *α*, *β*, *γ* > 0, 0 < *μ* < *α*, and 0 < *λ*_2_ < 1 (*λ*_2_ < 1 ensures a two-species food-chain) and finally the chemostat estimate

(19) $$\begin{array}{c} 0\leq \kappa <\gamma . \end{array}$$

The case *κ* = 0 corresponds to the known case *b* = 0 (see Lindström and Cheng [8]) and the main purpose of this paper is to derive a Rosenzweig-MacArthur [10] criterion for (18) when 0 < *κ* < *γ*. For the variables we assume *ξ* ≥ 0, *η* ≥ 0.

We notice that the alternative transformations *x* = *mCξ*, *y* = *MmCη*, *τ* = *Dt*, *m*_1_ = *m*/*Db*, *m*_2_ = *M*/*DB*, *a*_1_ = 1/*abC*, *a*_2_ = 1/*ABmC* give system (1.2) in Kuang [6]. Therefore, the system under study is the same but the set of feasible parameters might be differently identified. Our reparametrization is more complicated. However, the properties of (3) suggest transformation (16) since formula (16) provides a normalization of the growth interval for *x* in (9) and the interval

(20) $$\begin{array}{c} \left[\;0,\frac{M\left(amC\left(1-D/\left(m/b\right)\right)-D\right)}{a\left(1-D/\left(m/b\right)\right)}\;\right] \end{array}$$

for *y* into the unit interval for both variables *ξ* and *η*.

## 4. Isocline Form and Properties of Equilibria

We rewrite the system on a form allowing isoclines to be analyzable. It is far from clear how this should be done in the chemostat case. We decided to work with the following form:

(21) ξ˙=fξρξHξ+η−η,η˙=ηψξ,

and state our conditions on the involved functions as

(C-I) *f*, *ψ*, *ρ*, and *H* are *C* ^1^([0, *∞*)),
(C-II) *f*(0) = 0, *f*(*ξ*) > 0 for *ξ* > 0,
(C-III) *H*′(*s*) < 0, *H*(1) = 0,
(C-IV) (*ξ* − *λ*_2_)*ψ*(*ξ*) > 0, *ξ* ≠ *λ*_2_ > 0,
(C-V) *ρ*(*ξ*) > 0, *ρ*′(*ξ*) > 0, *ξ* > 0,
(C-VI) −*f*(*ξ*) + *ψ*(*ξ*) < 0, *λ*_2_ < *ξ* ≤ 1.

We note that system (18) corresponds to the choice

(22) fξ=αξ1+βξ,ρξ=1+βξα,Hs=1−s1+γ−κs,ψξ=μξ−λ21+βξ

and that this choice meets conditions (C-I)–(C-VI). In particular, we have (C-III) since

(23) $$\begin{array}{c} H^{\prime }\left(\;s\;\right)=-\frac{1+\gamma -\kappa }{{\left(1+\gamma -\kappa s\right)}^{2}}<0 \end{array}$$

and (C-IV) since

(24) −fξ+ψξ−αξ1+βξ+μξ−λ21+βξ=μ−αξ−μλ21+βξ<0

and the last inequality holds simply because *α* > *μ*. Before going further, we prove a basic theorem.

**Theorem 1 .**

Consider the bounded set *ξ* ≥ 0, *η* ≥ 0, *ξ* + *η* ≤ 1. Solutions of (21) starting in this set remain there.

**Proof**

By uniqueness of solutions no solutions can intersect the four solutions *ξ* = 0, 0 < *η* ≤ 1, *η* = 0, 0 < *ξ* < 1, (*ξ*, *η*) = (0,0), and (*ξ*, *η*) = (1,0). Thus, solutions remain positive. To prove that solutions remain bounded, we assume 1 ≤ *ξ* + *η* ≤ *γ*/*κ* and consider the series of inequalities

(25) ξ˙+η˙fξρξHξ+η−η+ηψξ≤−fξ+ψξη<0.

We further conclude that

(26) $$\begin{array}{c} H^{\prime \prime }\left(\;s\;\right)=-2\kappa \frac{1+\gamma -\kappa }{{\left(1+\gamma -\kappa s\right)}^{3}}<0, \end{array}$$

so we work with *H* decreasing and concave down as far as possible, too. We have one equilibrium at the origin, one at the carrying capacity (1,0), and one equilibrium at (*λ*_2_, *η*_*∗*_), where *η*_*∗*_ satisfies the condition *η*_*∗*_ = *ρ*(*λ*_2_)*H*(*λ*_2_ + *η*_*∗*_). We prove first, that the first two equilibria are saddles. The corresponding Jacobians are given by

(27) J0,0=f′0ρ0H000ψ0=11+γ00−μλ2,

(28) J1,0=f1ρ1H′1f1ρ1H′1−f10ψ1=−11+γ−κ−11+γ−κ−α1+β0μ1−λ21+β.

We note that *J*(1,0) also contains the information that the eigenvector corresponding to the positive eigenvalue points into the triangle *ξ* ≥ 0, *η* ≥ 0, and *ξ* + *η* ≤ 1. This criterion can be formulated as

(29) $$\begin{array}{c} 0<\frac{-H^{\prime }\left(1\right)+\psi \left(1\right)}{-H^{\prime }\left(1\right)+f\left(1\right)}<1, \end{array}$$

which is true due to *α* > *μ* and *f*(1) > *ψ*(1) > 0.

## 5. Implicit Functions and Our Criterion

For the interior equilibrium (*λ*_2_, *η*_*∗*_) we start by doing some estimates concerning its location and define an implicit function $\tilde{\eta }$ for the prey isocline by the equation

(30) $$\begin{array}{c} \rho \left(\;\xi \;\right)H\left(\;\xi +\tilde{\eta }\left(\;\xi \;\right)\;\right)-\tilde{\eta }\left(\;\xi \;\right)=0. \end{array}$$

By the implicit function theorem, we get

(31) $$\begin{array}{c} {\tilde{\eta }}^{\prime }\left(\;\xi \;\right)=\frac{\rho^{\prime }\left(\xi \right)H\left(\xi +\tilde{\eta }\left(\xi \right)\right)+\rho \left(\xi \right)H^{\prime }\left(\xi +\tilde{\eta }\left(\xi \right)\right)}{1-\rho \left(\xi \right)H^{\prime }\left(\xi +\tilde{\eta }\left(\xi \right)\right)}. \end{array}$$

The denominator of the above expression is always positive and therefore the implicit function is defined for 0 ≤ *ξ* ≤ 1. The sign of the derivative of the implicit function $\tilde{\eta }(\xi )$ is defined by the nominator. We start computing special values of this implicit function and conclude that $\tilde{\eta }(1)=0$ and

(32) $$\begin{array}{c} -1<\frac{\rho \left(1\right)H^{\prime }\left(1\right)}{1-\rho \left(1\right)H^{\prime }\left(1\right)}={\tilde{\eta }}^{\prime }\left(\;1\;\right)<0. \end{array}$$

We now go on computing the Jacobian of (21) at the interior equilibrium (*λ*_2_, *η*_*∗*_) and get 

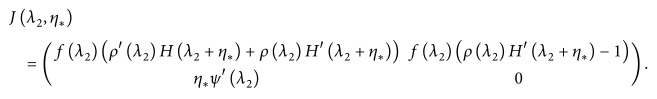
(33)We have that

(34) det Jλ2,η∗=−η∗ψ′λ2fλ2ρλ2H′λ2+η∗−1>0,

so the eigenvalues have the same sign and their stability is determined by the trace only. This is entirely in concordance with index theory [12, 13] asserting that the index of all fixed points in the interior is 1. We have

(35) Tr Jλ2,η∗=fλ2·ρ′λ2Hλ2+η∗+ρλ2H′λ2+η∗

and conclude that the sign of this expression agrees with the sign of (31). Thus, we have a Rosenzweig-MacArthur [10] criterion for the chemostat. We summarize our conclusions in the following theorem.

**Theorem 2 (a Rosenzweig-MacArthur [10] graphical criterion for the chemostat).**

Assume (C-I)–(C-VI). The interior fixed point of (21) is locally stable when ${\tilde{\eta }}^{\prime }(\lambda_{2})<0$ and unstable when ${\tilde{\eta }}^{\prime }(\lambda_{2})>0$. When ${\tilde{\eta }}^{\prime }(\lambda_{2})>0$, the chemostat system has at least one limit cycle. The prey isocline $\tilde{\eta }(\xi )$ decreases at the vicinity of 1 and is located in the bounded set *ξ* ≥ 0, *η* ≥ 0, *ξ* + *η* ≤ 1 for all 0 ≤ *ξ* ≤ 1.

**Figure 2:**
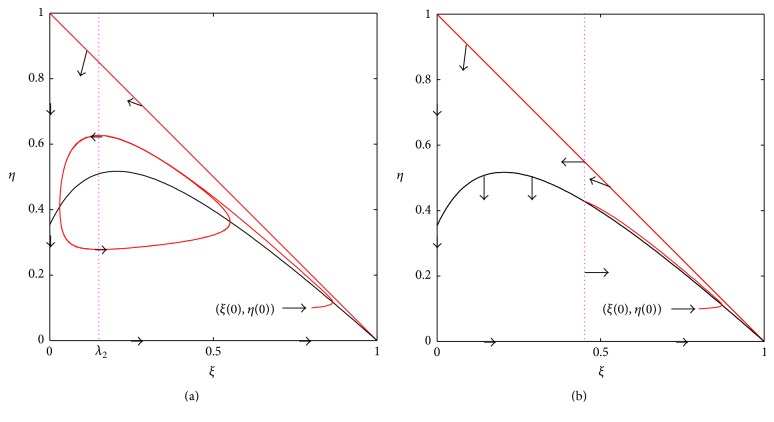
(a) System (21) has at least one limit cycle if ${\tilde{\eta }}^{\prime }(\lambda_{2})>0$ and (C-I)–(C-VI). The interior fixed point of system (21) is locally asymptotically stable when ${\tilde{\eta }}^{\prime }(\lambda_{2})<0$ and (C-I)–(C-VI). Our prototype example (18) is indeed globally asymptotically stable if ${\tilde{\eta }}^{\prime }(\lambda_{2})<0$ [1].

The last assertion is due to the Poincaré-Bendixson theorem (see, e.g., [9]) because the triangular set *ξ* > 0, *η* > 0, *ξ* + *η* < 1 is invariant and its boundary is not approached by any of the solutions. We illustrate the graphical conclusions of Theorem 2 in Figure 2.

Finally, we return to the specific expressions that we used as our prototype example (18) in order to check what additional conclusions can be made. We note that

(36) $$\begin{array}{c} 1-\left(\;1+\alpha +\gamma \alpha \;\right)\tilde{\eta }\left(\;0\;\right)+\alpha \kappa \tilde{\eta }{\left(\;0\;\right)}^{2}=0 \end{array}$$

has one solution in the unit interval and one solution greater than 1 (insert *η*(0) = 0 and *η*(0) = 1, resp., in the above equation and remember the chemostat estimate (19)). We are interested in the solution in the unit interval and it turns out to be

(37) $$\begin{array}{c} \tilde{\eta }\left(\;0\;\right)=\frac{1+\alpha +\gamma \alpha -\sqrt{{\left(1+\alpha +\gamma \alpha \right)}^{2}-4\alpha \kappa }}{2\alpha \kappa }. \end{array}$$

We also conclude that

(38) −1<η~′1=−1+βα+αγ−κ+1+β<0,η~′0=−1−γ+κ+β1−η~01+γ−κη~01+γ−κ+α1+γ−κη~02

because of (19). We continue with an analysis of the shape of this curve. The defining equation (30) can be written as

(39) $$\begin{array}{c} \frac{1+\beta \xi }{\alpha }\cdot \frac{1-\xi -\eta }{1+\gamma -\kappa \left(\xi +\eta \right)}=\eta , \end{array}$$

which after some refinement is the level curve of a quadratic form given by

(40) 1+βξ+1+α+γαη+βξ2+β−καξη−καη2=1.

We note immediately that the quadratic form at the right hand side must be indefinite (both leading terms +*βξ* ^2^ and −*καη* ^2^ are of different sign). The level curve consists therefore of either two intersecting lines or a hyperbola. One branch of this hyperbola is the predator isocline curve $\eta =\tilde{\eta }(\xi )$ and when the involved functions are given as in (22), the isocline curve is given explicitly by

(41) $$\begin{array}{c} \tilde{\eta }\left(\;\xi \;\right)=\frac{1+\beta \xi +\alpha +\alpha \left(\gamma -\kappa \xi \right)-\sqrt{{\left(1+\beta \xi +\alpha +\alpha \left(\gamma -\kappa \xi \right)\right)}^{2}-4\kappa \alpha \left(1+\beta \xi \right)\left(1-\xi \right)}}{2\kappa \alpha }. \end{array}$$

The minus sign was selected in front of the square root above since we require $\tilde{\eta }(1)=0$. We conclude that this isocline is concave down and ${\tilde{\eta }}^{\prime }(1)<{\tilde{\eta }}^{\prime }(0)$.

**Theorem 3 .**

The prey isocline $\tilde{\eta }(\xi )$ for our chemostat system (21) with (22) is a concave down function corresponding to a branch of a hyperbola.
